# Supplementary material for: Membrane protein MHZ3 regulates the on-off switch of ethylene signaling in rice
Source: Nat Commun. 2024 Jul 16;15:5987. doi: 10.1038/s41467-024-50290-4 (PMC11252128; doi:10.1038/s41467-024-50290-4)
Supplement: Supplementary file 1 — Supplementary Information [file 41467_2024_50290_MOESM1_ESM.pdf]

Membrane protein MHZ3 regulates the on-off switch of ethylene signaling in rice

Xin-Kai Li[1,2], Yi-Hua Huang[1], Rui Zhao[1], Wu-Qiang Cao[1], Long Lu[3], Jia-Qi Han[1], Yang Zhou[1], Xun Zhang[1,2], Wen-Ai Wu[1,2], Jian-Jun Tao[1], Wei Wei[1], Wan-Ke Zhang[1], Shou-Yi Chen[1], Biao Ma[4], He Zhao[5]*, Cui-Cui Yin[1]*, Jin-Song Zhang[1,2]*

***Authors for correspondence:**

Jin-Song Zhang

Email: jszhang@genetics.ac.cn;

Cui-Cui Yin

Email: ccyin@genetics.ac.cn;

He Zhao

Email: He.Zhao@tsl.ac.uk

This PDF file includes:

Supplementary Figures 1-17
Supplementary Table 1

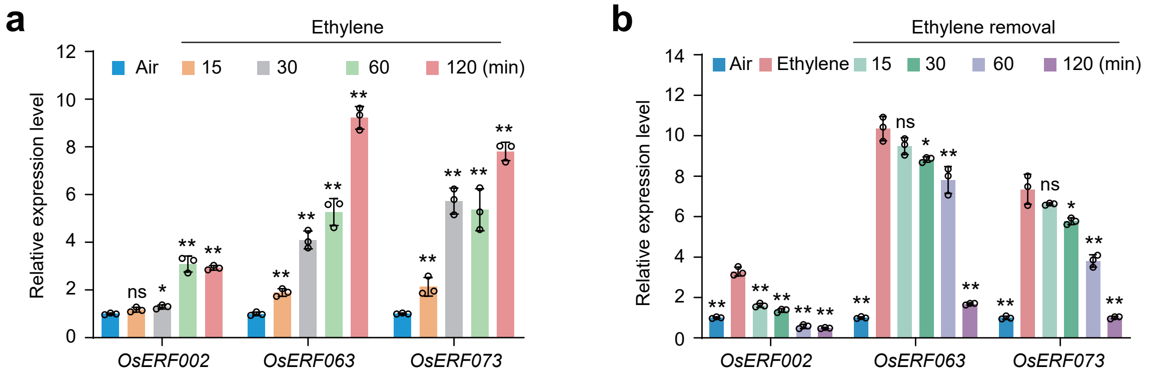

Supplementary Fig. 1 | Expression of ethylene-responsive genes during ethylene treatment and recovery. **a** Expression of ethylene-responsive genes was increased in response to ethylene. Total RNA extracted from the coleoptiles of 2-day-old etiolated seedlings, treated with 10 μL/L ethylene for varying durations, was analyzed by quantitative reverse transcription polymerase chain reaction (qRT-PCR). Data are means ± SD, n = 3 biological replicates (ns, not-significant; **P* < 0.05; ***P* < 0.01; two-tailed Student's *t*-test; compared to the corresponding Air). **b** Ethylene-induced expression of ethylene-responsive genes gradually decreased after ethylene removal. Etiolated seedlings of the wild type were treated with ethylene for 120 min. Subsequently, ethylene was removed and samples were taken at different time points. Total RNA from the coleoptiles was then subjected to qRT-PCR analysis. Data are means ± SD, n = 3 biological replicates (ns, not-significant; **P* < 0.05, ***P* < 0.01; two-tailed Student's *t*-test; compared to the corresponding Ethylene). Source data is in the Source Data file.

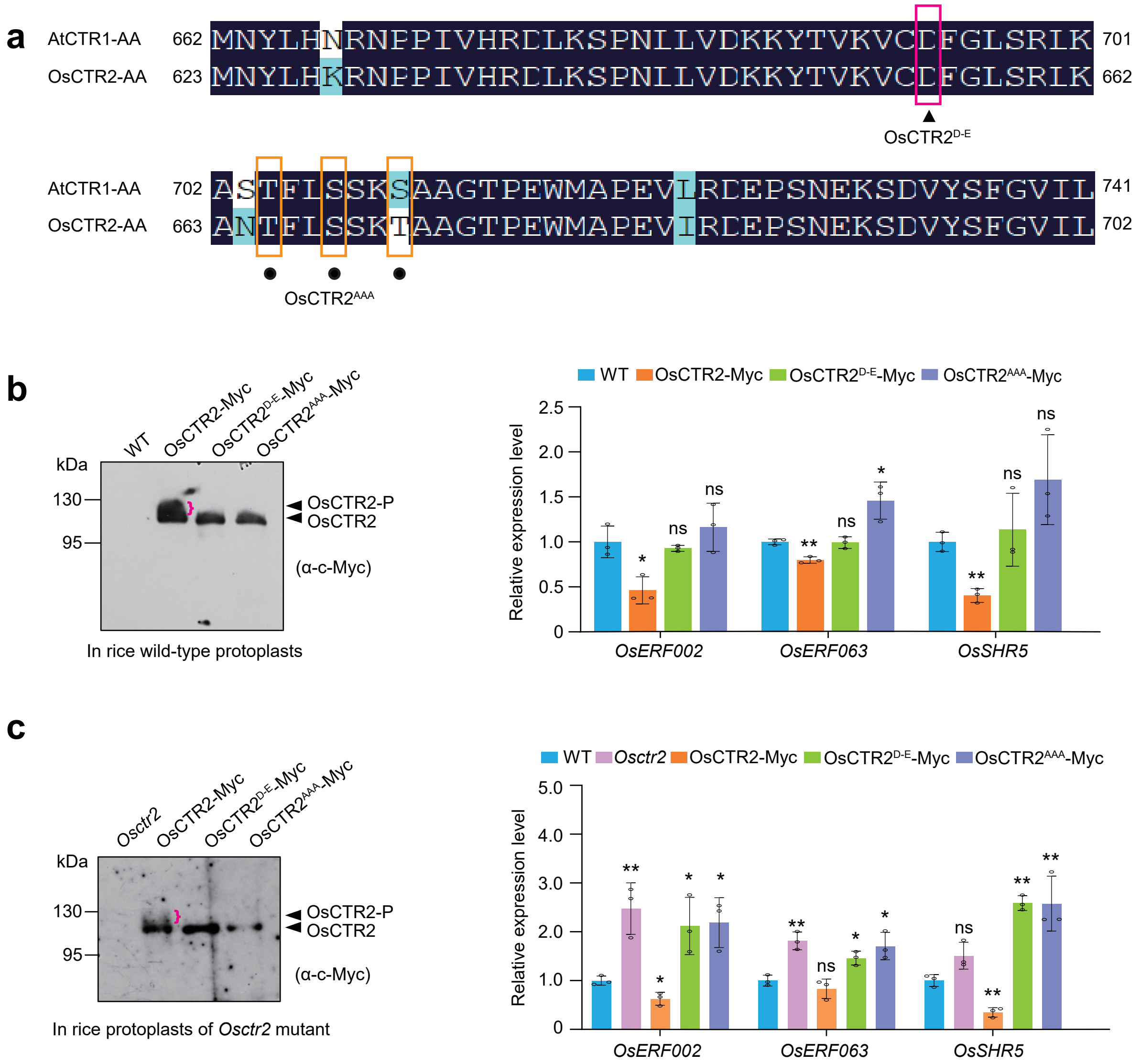

Supplementary Fig. 2 | The autophosphorylation of OsCTR2 is crucial for its functionality. a Partial amino acid sequences of OsCTR2 were compared with those of Arabidopsis AtCTR1. A triangle indicates the substitution site of OsCTR2$^{D\text{-}E}$, which imitates the mutant version AtCTR1^{D694E} of Arabidopsis AtCTR1-1, rendering it catalytically inactive. A dot indicates the substitution site of OsCTR2^{AAA}, which mimics the AtCTR1$^{T704/S707/S710\text{-}AAA}$ mutant form, leading to the loss of AtCTR1's ability for autophosphorylation and homodimerization. **b** In wild-type (WT) protoplasts, both OsCTR2$^{D\text{-}E}$ and OsCTR2^{AAA} are unable to undergo autophosphorylation and lose their ability to inhibit ethylene-responsive genes. **c** In *Osctr2* protoplasts, both OsCTR2$^{D\text{-}E}$ and OsCTR2^{AAA} are unable to undergo autophosphorylation and lose their ability to inhibit ethylene-responsive genes. In **b** and **c**, the *OsCTR2-Myc*, *OsCTR2$^{D\text{-}E}$-Myc*, and *OsCTR2^{AAA}-Myc* plasmids were expressed separately in WT and *Osctr2* protoplasts. The proteins were detected using an anti-c-Myc antibody. Red half-brackets indicate the phosphorylated form of OsCTR2. Data are means ± SD, n = 3 biological replicates (ns, not-significant; *P < 0.05, **P < 0.01; two-tailed Student's t-test; compared to the corresponding WT). Three independent experiments were repeated with similar results. Uncropped blots and source data are in the Source Data file.

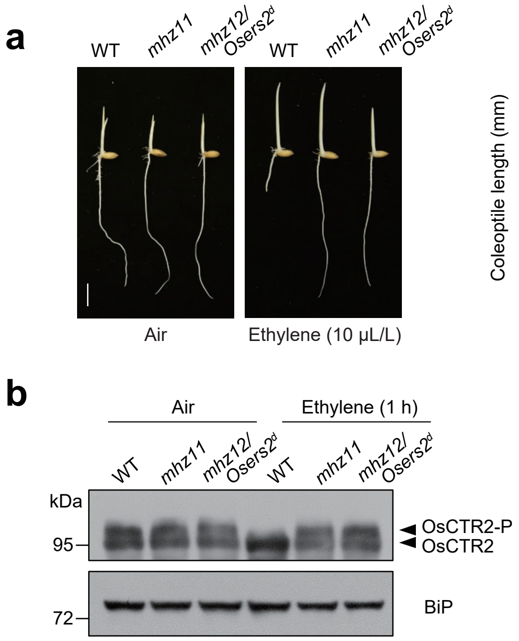

Supplementary Fig. 3 | Ethylene response phenotype of the *mhz11* and *mhz12/Osers2ᵈ* mutants and the phosphorylation state of OsCTR2 in these mutants. a Ethylene response phenotype of *mhz11* and *mhz12/Osers2ᵈ*. Etiolated seedlings of WT, *mhz11*, and *mhz12/Osers2ᵈ* were treated with or without 10 μL/L ethylene for 2.5 days. Coleoptile and root lengths are means ± SD, n = 30 biologically independent plants (***P* < 0.01; two-tailed Student's *t*-test; compared to the corresponding WT). Scale bar = 10 mm. **b** The phosphorylation state of OsCTR2 in *mhz11* and *mhz12/Osers2ᵈ*. Etiolated seedlings of 2-day-old were treated with 10 μL/L ethylene for 1 hour. Anti-OsCTR2 antibody was used to detect the phosphorylation state of OsCTR2, with BiP (an ER membrane marker) serving as the internal reference. Two independent experiments were repeated with similar results. Uncropped blots and source data are in the Source Data file.

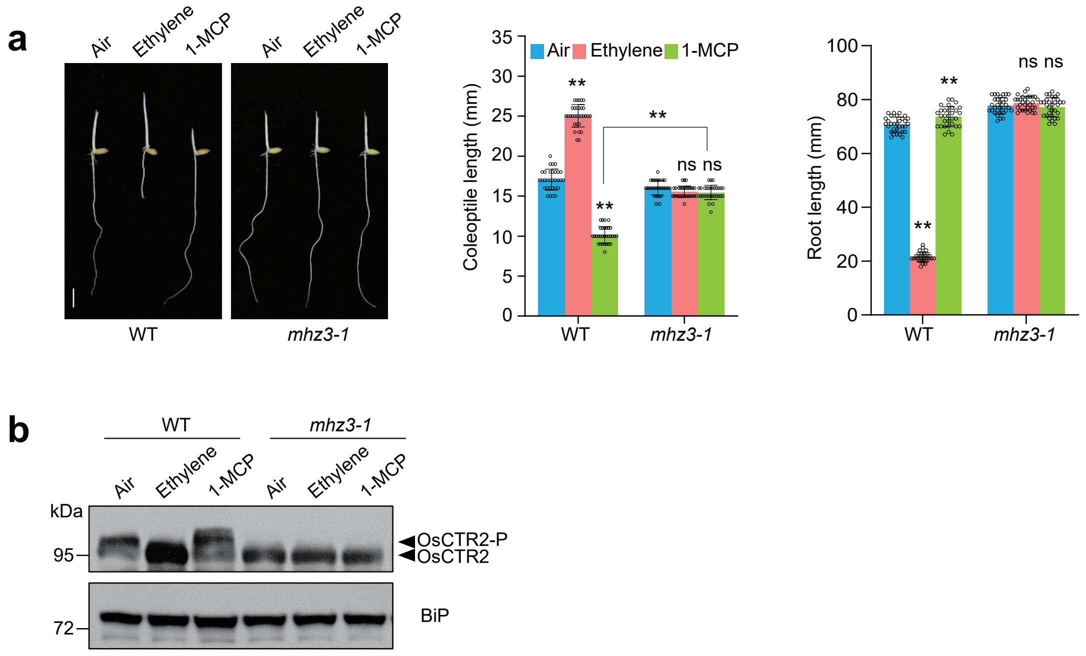

Supplementary Fig. 4 | Ethylene and 1-MCP response phenotype and the phosphorylation state of OsCTR2 in *mhz3-1*. a *mhz3-1* is insensitive to ethylene and 1-MCP. Etiolated seedlings grown in air, 10 µL/L ethylene, or 10 µL/L 1-MCP for 3 days were shown, scale bar = 10 mm. Coleoptile and root lengths are means ± SD, n = 30 biologically independent plants (ns, not-significant; ***P* < 0.01; two-tailed Student's *t*-test; compared with the corresponding Air). **b** The phosphorylation state of OsCTR2 in *mhz3-1*. Etiolated seedlings of 2-day-old dark-grown seedlings were treated with air, 10 µL/L ethylene, or 10 µL/L 1-MCP for 1 hour. After treatment, the seedlings were collected for protein extraction. Anti-OsCTR2 antibody was used to detect the phosphorylation state of OsCTR2, with BiP (an ER membrane marker) serving as the internal reference. 1-MCP: 1-methylcyclopropene. Two independent experiments were repeated with similar results. Uncropped blots and source data are in the Source Data file.

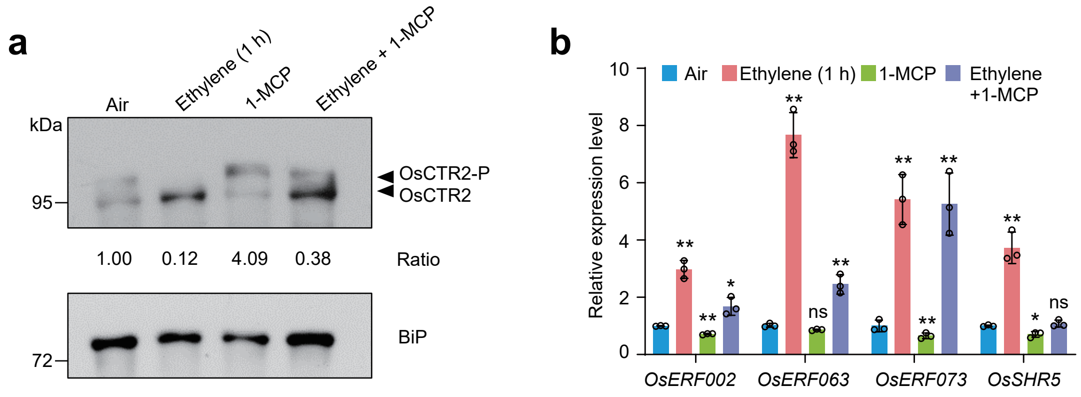

Supplementary Fig. 5 | The exogenous application of the ethylene receptor inhibitor 1-MCP resulted in elevated OsCTR2 phosphorylation and suppressed the activation of ethylene signaling. a Treatment with 10 μL/L 1-MCP enhances the phosphorylation of OsCTR2. Ratio represents the ratio of OsCTR2-P to OsCTR2. The phosphorylation state of OsCTR2 was detected using the anti-OsCTR2 antibody, with BiP (an ER membrane marker) serving as the internal reference. **b** 1-MCP suppresses the expression of ethylene-responsive genes. Data are means ± SD, n = 3 biological replicates (ns, not-significant; *$P < 0.05$, **$P < 0.01$; two-tailed Student's *t*-test; compared to the corresponding Air). For **a** and **b**, wild-type (WT) etiolated seedlings grown for 2 days were treated with 10 μL/L ethylene, 10 μL/L 1-MCP, and a combination of 10μL/L ethylene and 1-MCP for 1 hour. 1-MCP: 1-methylcyclopropene. Three independent experiments were repeated with similar results. Uncropped blots and source data are in the Source Data file.

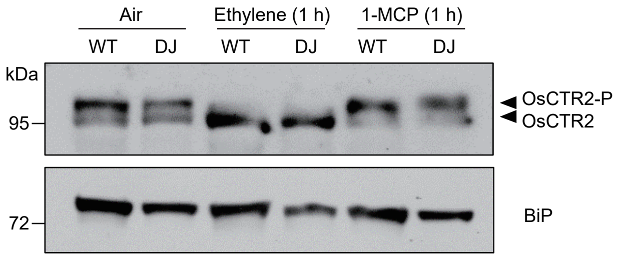

Supplementary Fig. 6 | The phosphorylation of OsCTR2 in both Nipponbare (WT) and Dongjin (DJ) is similar in response to ethylene and 1-MCP. Two-day-old etiolated seedlings were exposed to air, 10 µL/L ethylene, or 10 µL/L 1-MCP for 1 hour prior to being collected for protein extraction. Anti-OsCTR2 antibody was utilized to detect the phosphorylation state of OsCTR2, with BiP (an ER membrane marker) serving as the internal reference. Three independent experiments were repeated with similar results. Uncropped blots in Source Data file.

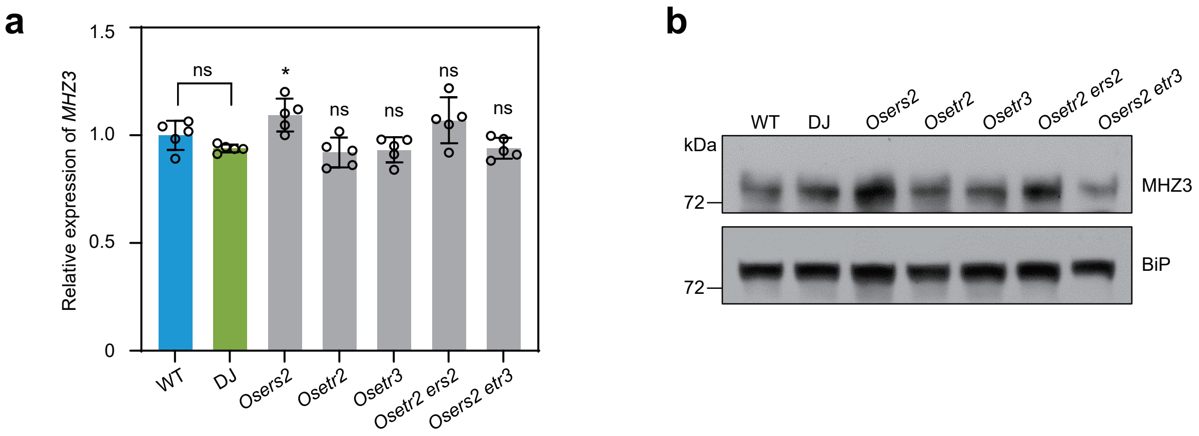

Supplementary Fig. 7 | *MHZ3* expression level and protein content in the ethylene receptor mutants. a Expression of *MHZ3* in different ethylene receptor mutants. Statistical analysis of the relative expression of *MHZ3* is presented, and the data are means ± SD, n = 5 biological replicates (ns, not-significant; *$P < 0.05$; two-tailed Student's *t*-test; compared with DJ). **b** MHZ3 protein content in different ethylene receptor mutants. Total proteins were immunoblotted with anti-MHZ3 antibody, with BiP serving as an internal reference. DJ: Dongjin. Two independent experiments were repeated with similar results. Uncropped blots and source data are in the Source Data file.

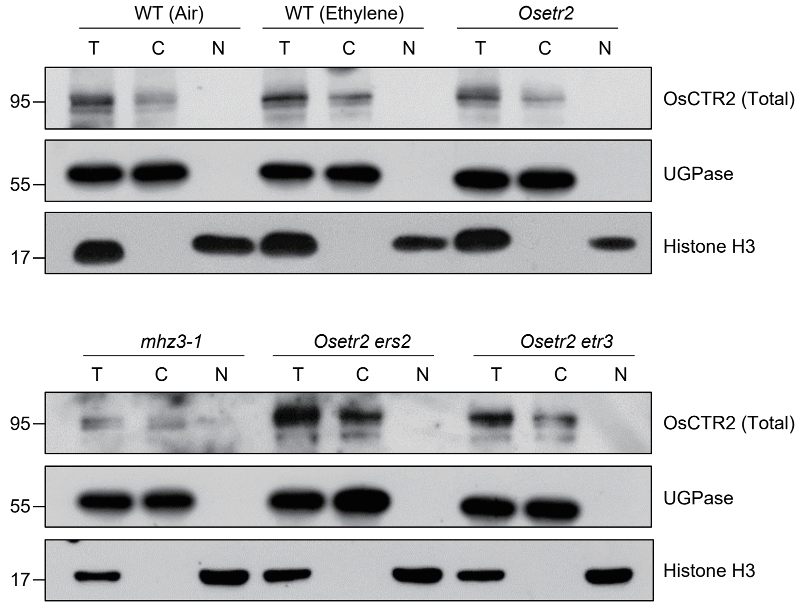

Supplementary Fig. 8 | Fractionation analysis of OsCTR2 isolated from various etiolated seedlings. Total protein extracts (T) from WT (Air), WT (10 µL/L Ethylene, 1 hour), *Osetr2*, *mhz3-1*, *Osetr2 ers2*, and *Osetr2 etr3* etiolated seedlings were fractionated into cytoplasmic (C) and nuclear (N) fractions, and subjected to immunoblotting with anti-OsCTR2, anti-UGPase, and anti-Histone H3 antibodies**.** Three independent experiments were repeated with similar results. Uncropped blots in Source Data file.

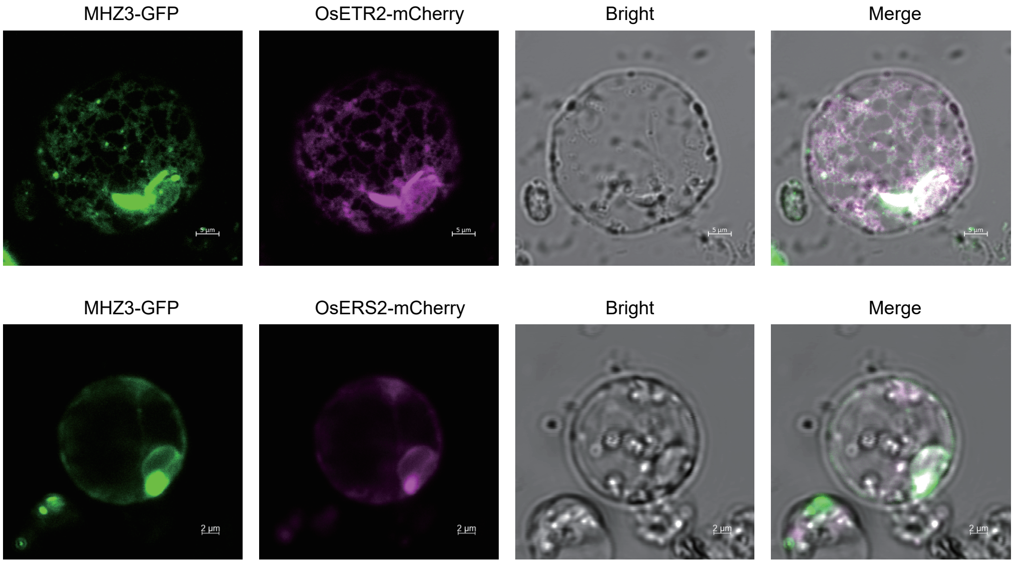

Supplementary Fig. 9 | MHZ3 co-localizes with OsETR2 and OsERS2 in rice protoplasts. The *MHZ3-GFP* plasmid was cotransformed with either *OsETR2-mCherry* or Os*ERS2-mCherry* plasmids into rice wild-type protoplasts.

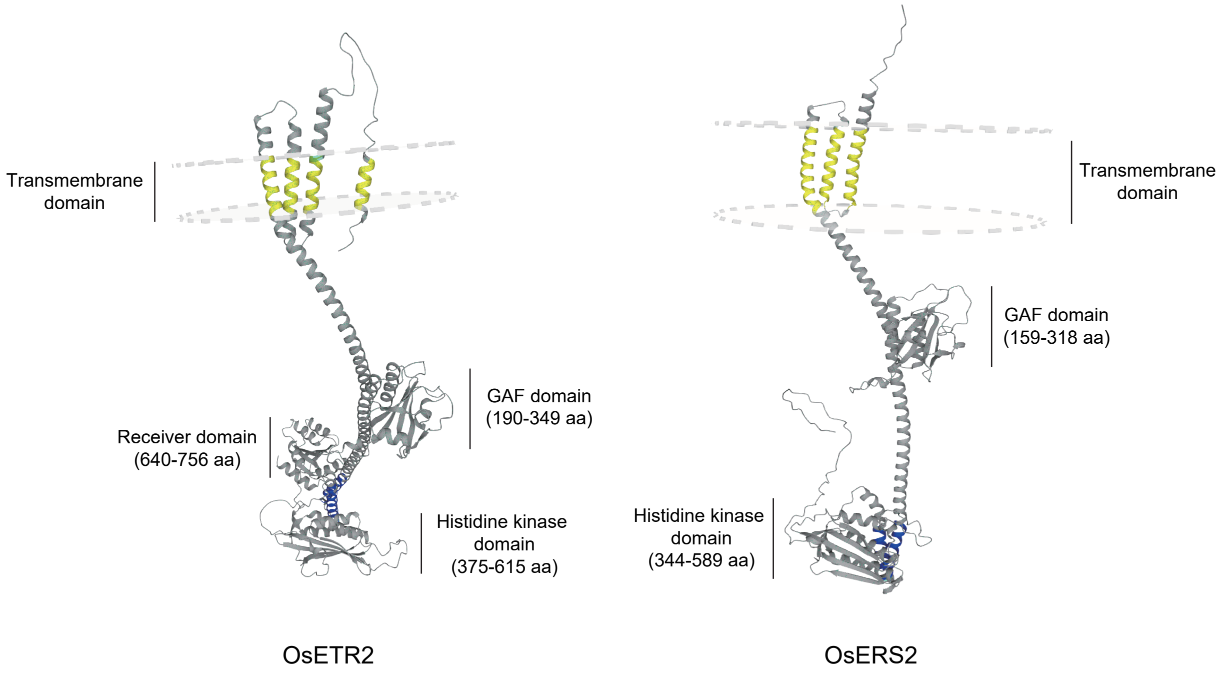

Supplementary Fig. 10 | Membrane-embedded form analysis of ethylene receptors OsETR2 and OsERS2 using TmAlphaFold. Utilize the TmAlphaFold database (https://tmalphafold.ttk.hu/) to predict the membrane-embedded form and 3D structure of the ethylene receptor proteins OsETR2 and OsERS2. GAF is short for cGMP-specific phosphodiesterases, Adenylyl cyclases, and FhlA.

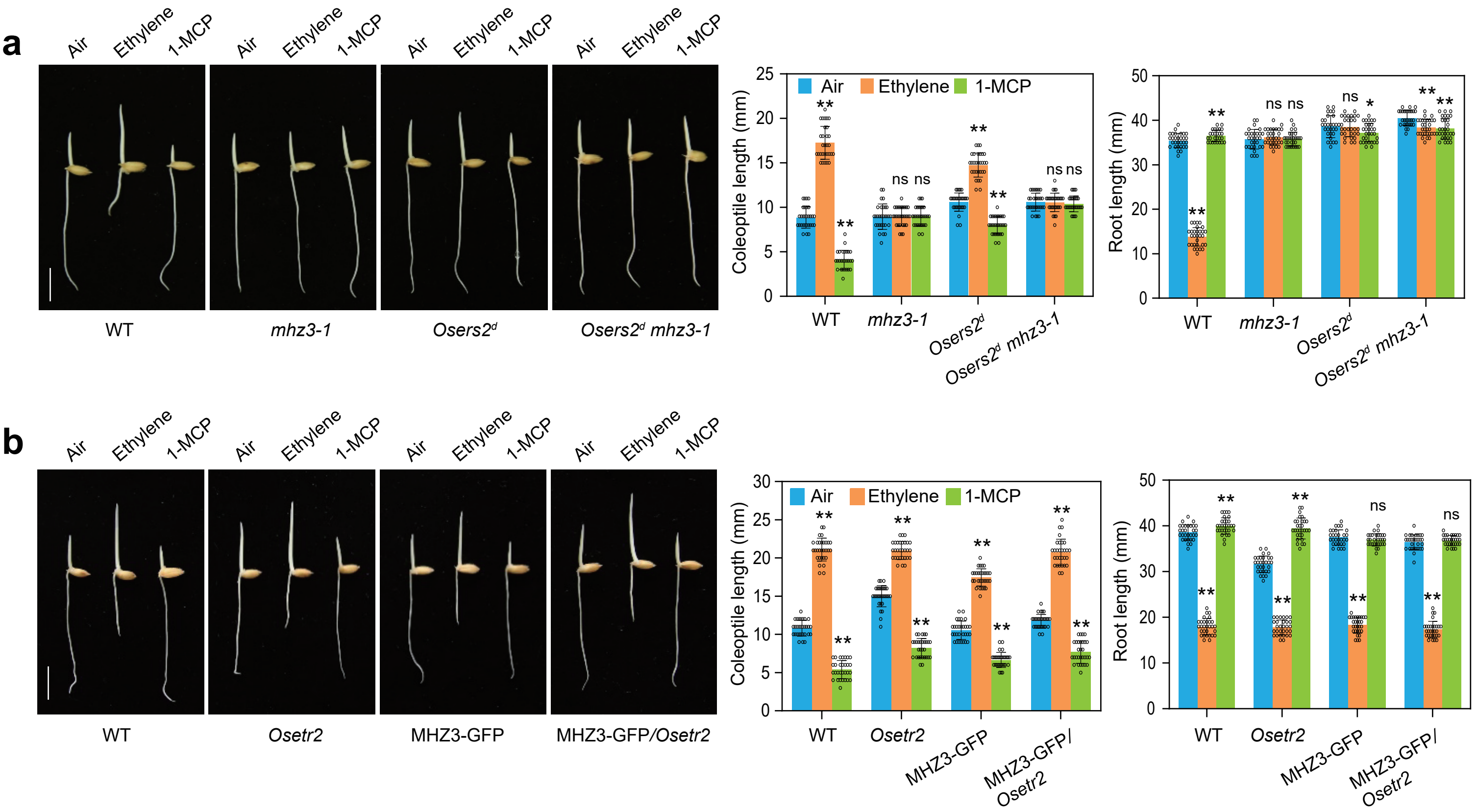

Supplementary Fig. 11 | The phenotypes of *Osers2^{d} mhz3-1* and MHZ3-GFP/*Osetr2*. a The phenotype of *Osers2^{d} mhz3-1*. Etiolated seedlings were treated with air, 10 μL/L ethylene, or 10 μL/L 1-MCP for 2 days. The lengths of coleoptiles and roots are shown as means ± SD, with n value exceeding 27 independent biological plants (ns, not-significant; **P* < 0.05, ***P* < 0.01; two-tailed Student's *t*-test; compared to the corresponding Air). **b** The phenotype of MHZ3-GFP/*Osetr2*. Etiolated seedlings were treated with air, 10 μL/L ethylene, or 10 μL/L 1-MCP for 2 days. The lengths of coleoptiles and roots are shown as means ± SD, n = 30 biologically independent plants (ns, not-significant; ***P* < 0.01; two-tailed Student's *t*-test; compared to the corresponding Air). Scale bar = 10 mm. Source data is in the Source Data file.

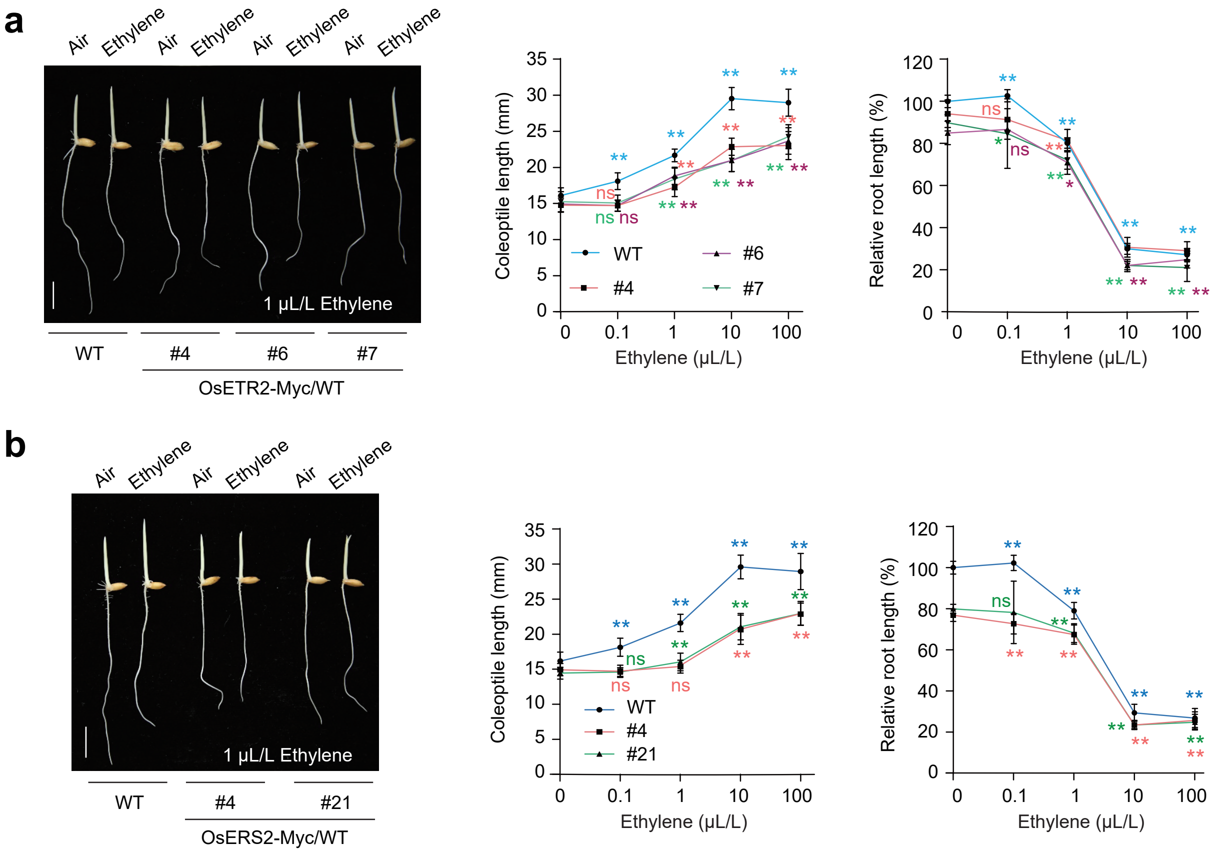

Supplementary Fig. 12 | Ethylene response phenotype of OsETR2-Myc and OsERS2-Myc overexpression plants in WT background. a Ethylene response phenotypes of OsETR2-Myc/WT transgenic lines (#4, #6 and #7). **b** Ethylene response phenotypes of OsERS2-Myc/WT transgenic lines (#4 and #21). Dark-grown seedlings of the wild-type (WT), OsETR2-Myc/WT (**a**), and OsERS2-Myc/WT (**b**) were treated with varied concentrations of ethylene for 3 days. Representative seedlings grown with or without 1 μL/L of ethylene are shown, scale bars = 10 mm. Coleoptile and relative root lengths are means ± SD, with n value exceeding 28 independent biological plants (ns, not-significant; **P* < 0.05, ***P* < 0.01; two-tailed Student's *t*-test; compared to the corresponding Air). Source data is in the Source Data file.

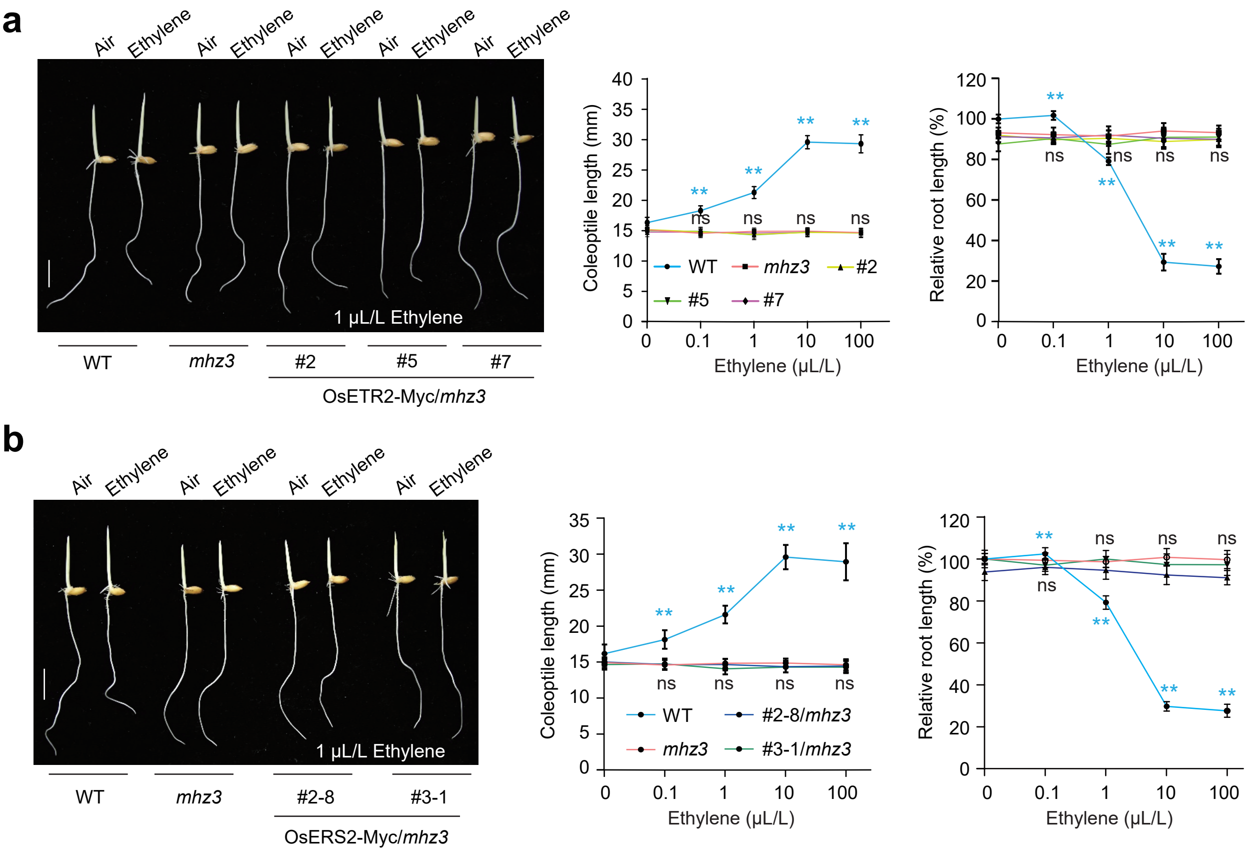

Supplementary Fig. 13 | Ethylene response phenotype of the plants overexpressing OsETR2-Myc or OsERS2-Myc in *mhz3*. a Ethylene response phenotypes of OsETR2-Myc/*mhz3* transgenic lines (#2, #5 and #7). **b** Ethylene response phenotypes of OsERS2-Myc/*mhz3* transgenic lines (#2-8 and #3-1). Dark-grown seedlings of the wild-type (WT), *mhz3*, OsETR2-Myc/*mhz3* (**a**), and OsERS2-Myc/*mhz3* (**b**) were treated with varied concentrations of ethylene for 3 days. Representative seedlings grown with or without 1 µL/L of ethylene are shown, scale bars = 10 mm. Coleoptile and relative root lengths are means ± SD, n = 30 biologically independent plants (ns, not-significant; **P < 0.01; two-tailed Student's t-test; compared to the corresponding Air). Source data is in the Source Data file.

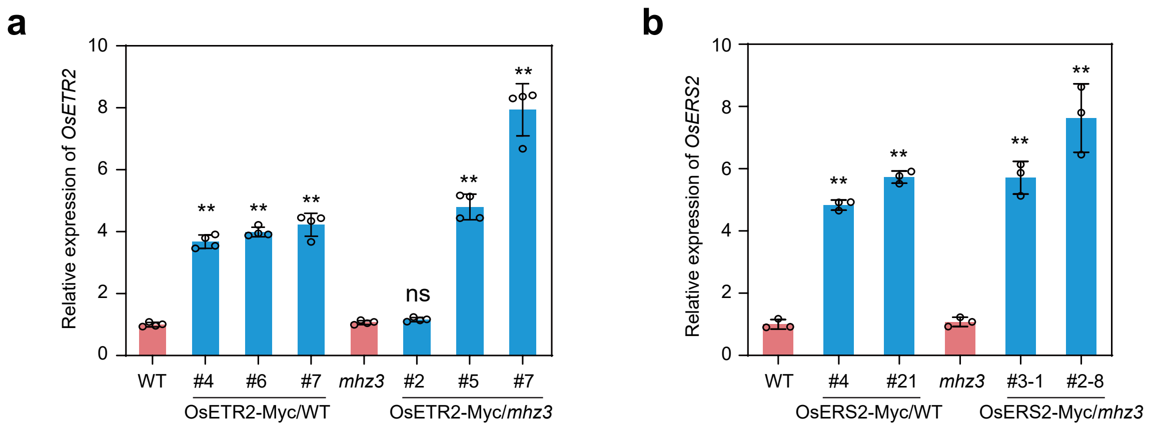

Supplementary Fig. 14 | Expression of OsETR2 and OsERS2 in OsETR2-Myc and OsERS2-Myc transgenic plants. **a** Expression of *OsETR2* in OsETR2-Myc/WT and OsETR2-Myc/*mhz3* transgenic lines. **b** Expression of *OsERS2* in OsERS2-Myc/WT and OsERS2-Myc/*mhz3* transgenic lines. The data are shown as means ± SD, with n = 4 (**a**) and n = 3 (**b**) biological replicates (ns, not-significant; $**P < 0.01$; two-tailed Student's *t*-test; compared with WT or *mhz3*). Source data is in the Source Data file.

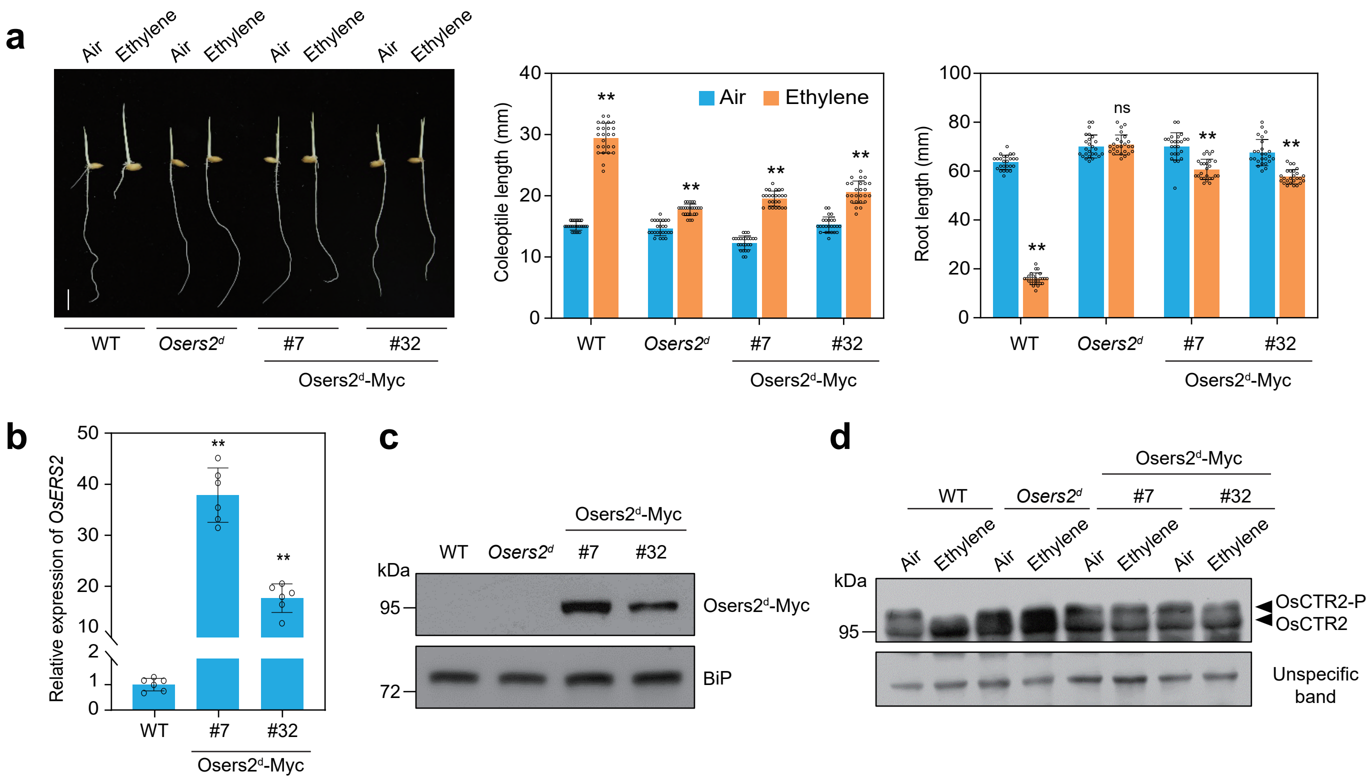

Supplementary Fig. 15 | Overexpressing Osers2ᵈ-Myc in WT mimics the ethylene-insensitive phenotype of *Osers2ᵈ*. a Ethylene response phenotype of Osers2ᵈ-Myc/WT. Etiolated seedlings of WT and Osers2ᵈ-Myc/WT were treated with or without 10 μL/L ethylene for 3 days, scale bars = 10 mm. Coleoptile and root lengths are means ± SD, n = 25 biologically independent plants (ns, not-significant; $^{**}P < 0.01$; two-tailed Student's *t*-test; compared to the corresponding Air). **b** Expression of *OsERS2* in Osers2ᵈ-Myc/WT transgenic lines. Statistical analysis of the relative expression of *OsERS2* is presented, and the data are shown as means ± SD, n = 6 biological replicates ($^{**}P < 0.01$; two-tailed Student's *t*-test; compared with WT). **c** Protein content of Osers2ᵈ-Myc in Osers2ᵈ-Myc/WT transgenic lines. The total protein was extracted from etiolated seedlings grown in the dark for 2 days. The protein content of Osers2ᵈ-Myc was detected using anti-c-Myc antibody. Bip was utilized as an internal reference. **d** The phosphorylation state of OsCTR2 in Osers2ᵈ-Myc. Etiolated seedlings of 2-day-old were treated with 10 μL/L ethylene for 1 hour. Anti-OsCTR2 antibody was used to detect the phosphorylation state of OsCTR2, and an unspecific band was utilized as an internal reference. Two independent experiments were repeated with similar results. Uncropped blots and source data are in the Source Data file.

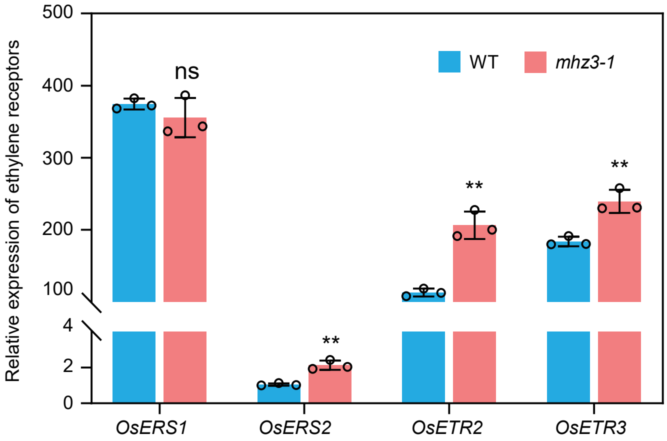

Supplementary Fig. 16 | Expression levels of ethylene receptor genes in WT and *mhz3-1*. Total RNAs from 2-day-old etiolated seedlings were subjected to qRT-PCR analysis. The data are means ± SD, n = 3 biological replicates (ns, not-significant; $^{**}P < 0.01$; two-tailed Student's *t*-test; compared with the corresponding WT). Source data is in the Source Data file.

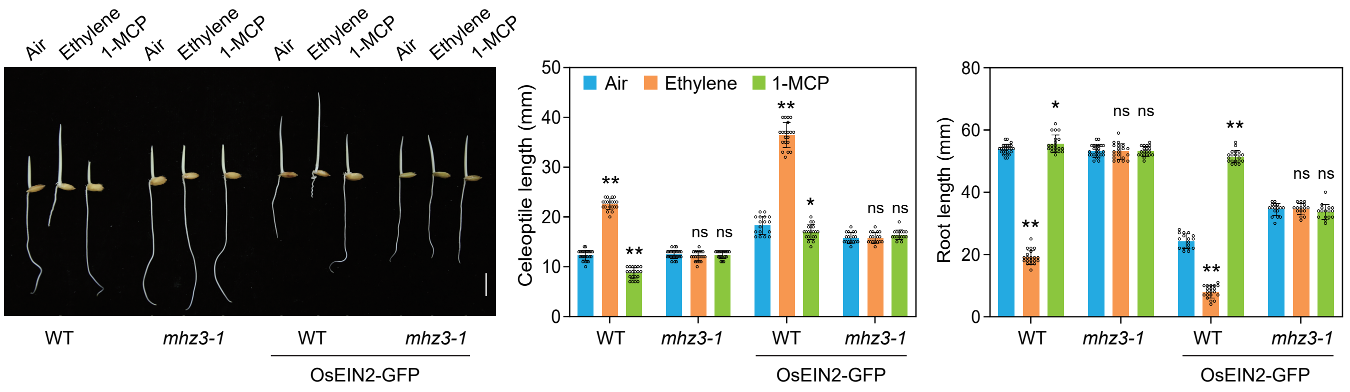

Supplementary Fig. 17 | Transgenic plants overexpressing OsEIN2 in the *mhz3-1* background exhibit no response to either ethylene or 1-MCP. Overexpression of OsEIN2 could not restore the ethylene and 1-MCP responses of *mhz3-1*. Etiolated seedlings were grown in air, 10 μL/L ethylene or 10 μL/L 1-MCP for 2.5 days. Coleoptile and root lengths are means ± SD, with n value exceeding 15 independent biological plants (ns, not-significant; **P* < 0.05, ***P* < 0.01; two-tailed Student's *t*-test; compared to the corresponding Air). Scale bar = 10 mm. 1-MCP: 1-methylcyclopropene. Source data is in the Source Data file.

Supplementary Table 1

Primers used in this study

Genes or constructs	Forward primer (5'-3')	Reverse primer (5'-3')
**qRT-PCR**		
MHZ3	tggtgagcgaggctatgttg	taggtccacacccctggtag
OsETR2	gttacttccggcatccaatg	tttgaaggactctatacagttc
OsERS2	acgcctaatggttgagactatac	gatggtagtcaggttcatgtg
OsUBQ5	tcaccaggctcaggaaggag	cagatcagagcaaagcgagc
OsERF002	gcagtacgtggaccagatgatc	ctcgatcagagttcttcctcac
OsERF063	acgtgatggacagcctcctc	gggaagtctgaaatggacatg
OsERF073	aatgataatcaaggcaccac	acccgaataagtgttgataac
OsSHR5	aataccagctatgttaccagcc	caccattacaaattacaaggagc
**Plasmid construction**		
Genetic transformation		
OsETR2-Myc	gctctagaatgccaccgatcccatctctgt	acgcgtcgacattgttttgaaggactctatacagt
OsERS2-Myc	cgggatccatggatggatcatgtgattgca	acgcgtcgactacgcttgattggtagcgaacc
OsCTR2-Myc<br>*OsCTR2*$^{D-E}$*-Myc*<br>*OsCTR2*^{AAA}*-Myc*	ggtacccggggatcctctagaatgaaggccgacgccaagtg	taattaacccgctgttatcactgatatcctcttgaagttg
Co-localization		
OsETR2-mCherry	gaggacagggtacccggggatccatgccaccgatcccatctct	tcctcgcccttgctcaccatattgttttgaaggactctat
OsERS2-mCherry	gaggacagggtacccggggatccatggatggatcatgtgattg	tcctcgcccttgctcaccattacgcttgattggtagcgaac
LCI		
Cluc-OsETR2	cgtcccggggcggtacctccatgccaccgatcccatctct	cctcagtcgacgcgttgtgtcaattgttttgaaggactc
Cluc-OsERS2	cgtcccggggcggtacctccatggatggatcatgtgattg	cctcagtcgacgcgttgtgtcatacgcttgattggtagcg
OsCTR2-Nluc	gagctcggtacctccggatccatgaaggccgacgccaag	gcctcagtcgacgcgttgtgactgatatcctcttgaagtt
MHZ3-Nluc	gagctcggtacctccggatccatgggcccaccacgtcgctc	gcctcagtcgacgcgttgtgatcaacatgatcatctac
GFP-Nluc	gacgagctcggtacctccggatccatggtgagcaagggcgag	gcgcctcagtcgacgcgttgtcttgtacagctcgtccatgc
Co-IP		
OsETR2-TMGAF-nYFP-FLAG	gacagggtacccggggatccatgccaccgatcccatctct	ttgctcaccatagaggatccctctcgcatcaattgagattc
OsETR2-GAFHR-nYFP-FLAG	gacagggtacccggggatccatggatcgccacacgatcctgt	ttgctcaccatagaggatccattgttttgaaggactctat
OsETR2-HR-nYFP-FLAG	gacagggtacccggggatccatggccactgaagctaggaact	ttgctcaccatagaggatccattgttttgaaggactctat
OsERS2-TMGAF-nYFP-FLAG	gacagggtacccggggatccatggatggatcatgtgattg	ttgctcaccatagaggatccatcacgggcccgcatggact
OsERS2-GAFHK-nYFP-FLAG	gacagggtacccggggatccatgctgacccatgagataaga	ttgctcaccatagaggatcctacgcttgattggtagcgaa
OsERS2-HK-nYFP-FLAG	gacagggtacccggggatccatggctatttgtgcccgtaat	ttgctcaccatagaggatcctacgcttgattggtagcgaa
MdYTH		
MHZ3-Cub	attaacaaggccattacggccgcggcggcggcggatacggag	aactgattggccgaggcggccccatcaacatgatcatctact
NubG-OsETR2	acggcccgggaaaaaacatgtatgccaccgatcccatctct	cgataagcttgatatcgaatttcaattgttttgaaggactc
NubG-OsERS2	acggcccgggaaaaaacatgtatggatggatcatgtgattg	cgataagcttgatatcgaatttcatacgcttgattggtagc
